# Creating person-al space for unspoken voices during diagnostic medical imaging examinations: a qualitative study

**DOI:** 10.1186/s12913-021-06958-4

**Published:** 2021-09-11

**Authors:** Chandra Rekha Makanjee, Anne-Marie Bergh, Deon Xu, Drishti Sarswat

**Affiliations:** 1 Department of Medical Radiation Science, University of Canberra, University Drive, Bruce, ACT 2617 Australia; 2 Research Centre for Maternal, Fetal, Newborn and Child Health Care Strategies, University of Pretoria, Private Bag X323, Gezina, 0083 South Africa

**Keywords:** Medical imaging, Patient voices, Patient experience, Vulnerability, Life-world, Service delivery quality

## Abstract

**Background:**

There is emerging interest in person-centred care within a short-lived yet complex medical imaging encounter. This study explored this event from the viewpoint of patients referred for an imaging examination, with a focus on the *person* and their *person-al* space.

**Methods:**

We used convenience sampling to conduct semi-structured interviews with 21 patients in a private medical imaging practice in Australia. The first phase of data analysis was conducted deductively, using the six elements of the person-centred, patient-journey framework of the Australian Commission on Safety and Quality in Healthcare: transition in; engagement; decisions; well-being; experience; and transition out. This was followed by inductive content analysis to identify overarching themes that span a patient’s journey into, through and out of an imaging encounter.

**Results:**

The *transition-in* phase began with an appointment and the first point of contact with the imaging department at reception. *Engagement* focused on patient-radiographer interactions and explanations to the patient on what was going to happen. *Decisions* related primarily to radiographers’ decisions on how to conduct a particular examination and how to get patient cooperation. Participants’ *well-being* related to their appreciation of gentle treatment; they also referred to past negative *experiences* that had made a lasting impression. *Transitioning out* of the imaging encounter included the sending of the results to the referring medical practitioner. *Person-al vulnerabilities* emerged as a cross-cutting theme. Patients’ vulnerability, for which they needed reassurance, pertained to uncertainties about the investigation and the possible results. Healthcare professionals were vulnerable because of patient expectations of a certain demeanour and of pressure to perform optimal quality investigations. Lastly, patients’ personal lives, concerns and pressures – their person-al ‘baggage’ – shaped their experience of the imaging encounter.

**Conclusion:**

To add value to the quality of the service they deliver, radiography practitioners should endeavour to create a person-al space for clients. Creating these spaces is complex as patients are not in a position to judge the procedures required by technical imaging protocols and the quality control of equipment. A reflective tool is proposed for radiographers to use in discussions with their team and its leaders on improving person-centred care and the quality of services in their practice.

## Background

Diagnostic medical imaging examinations play an important role in initiatives to improve public health [1]. The value lies in achieving a seamless continuum of high-quality care services and outcomes in a safe and timely manner [2, 3]. ‘Diagnosis, as both event and process, is central to the practices of contemporary medicine’ (p. 285) [4]. An imaging investigation is a brief temporal event contributing to the process followed by a medical practitioner in coming to a differential or final diagnosis. The only person who is present throughout all the interprofessional diagnostic events is the patient [5].

When referred for a diagnostic imaging investigation, a patient may already be in a vulnerable state [6]. From a technological viewpoint, practitioners have to perform technically intricate procedures and produce images of optimal quality [7, 8]. Their contribution to patient care and services is often driven by local service demands centred on the system [9, 10]. Patients, however, are more focused on human needs and expectations of establishing a meaningful trust relationship, characterised by the radiographer’s sincere interest and empathy [11]. The technological environment, painful and difficult procedures, the brevity of the encounter and power inequalities between practitioner and patient could result in patients feeling vulnerable and also that they are not being heard [4, 12].

The Australian Commission on Safety and Quality in Healthcare sees person-centred care (PCC) in healthcare service strategies and care models as a way to support the voice of the patient [13]. PCC, like patient-centred care and whole-person care, is a complex concept with many definitions and operationalisations [14]. The goal of PCC is to proactively create a space or environment that contributes to the meaningful life of a patient [10, 15]. Common requirements of PCC include: (1) coordinating the individualised care of patients within the health organisation; (2) engaging with patients to establish a relationship, working with them on what matters to them and sharing decision making; (3) using appropriate communication styles and making time to listen and share information; and (4) displaying attributes like empathy, respect and flexibility [10, 14–16]. Patients are valued for their lived experience and life stories and ‘the continuation of self and normality’ (p. 47) [17]. The outcomes of PCC for the patient include feelings of being understood, of well-being (decreased anxiety and fear), dignity, satisfaction and competence in making decisions [16, 18, 19].

PCC focuses on processes for delivering care through a range of activities that describe the actions and behaviours expected of nurses (and to a certain extent medical practitioners), often in longer-term care contexts [16, 20]. However, little is known about the feasibility or nature of PCC within the brief imaging encounter. The aim of this study was to explore patients’ experiences of a diagnostic medical imaging examination with a view to getting a sense of how spaces are created for their voice and how these spaces relate to the broader care environment and processes.

## Methods

A qualitative methodology was adopted to describe and understand how patient voices could be accommodated during a brief imaging encounter. A convenience sample of 22 participants visiting a private outpatient multimodality medical imaging practice in New South Wales, Australia, agreed to spend an extra half hour after their imaging investigation on an individual interview. They received verbal and written information on the study and their rights as participants and signed statements indicating informed consent. The audio-recorded interviews were conducted in a private room to ensure confidentiality. The semi-structured interview guide included questions on events that led to the imaging examination, expectations and experience of the imaging encounter and anticipation of the outcome of the examination. Interviews were conducted until data saturation was reached and no new information was being generated [21].

**Fig. 1:**
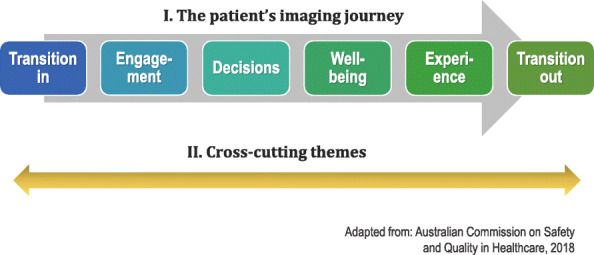
Data analysis framework

Audio-recordings were transcribed. The first two authors independently analysed the data manually and regularly discussed their findings to reach consensus, after which the other two authors provided further interpretation inputs. This allowed for investigator triangulation with varied perspectives to enhance the credibility and trustworthiness of the study. We used a two-pronged approach in the analysis (see Fig. 1). Firstly, we followed a deductive approach using the person-centred patient journey presented by the Australian Commission on Safety and Quality in Healthcare [13] to describe patient voices in the diagnostic imaging investigation space. This framework depicts the journey as having six key elements: transition in; engagement; decisions; well-being; experience; and transition out. The second data-analysis approach entailed an inductive content analysis [22] to identify categories or themes cutting across a *person*’s imaging journey from the perspective of creating *person*-al space. The unconventional hyphenation of ‘person-al’ is an attempt to keep the focus on the *person,* based on their ‘best interests’ and ‘quality of life’ (p. 56) [23] and to emphasise the human capabilities of patients as persons, which, according to Entwistle and Watt, include ‘person-al capabilities’ (p. 34) [25].

The Health Research Ethics Committee, Faculty of Health Sciences, University of Canberra, granted ethical approval for the study (Project 20180428) and the manager of the medical imaging practice gave written permission for the study to be conducted on site.

## Results

The 22 interviewees (11 male; 11 female) covered an age range of 20 to 78 years. Two participants were of Asian origin, two were Hispanic and the rest Caucasian. All participants referred for their current examination had had at least one previous examination such as a general radiographic examination, a computerised tomography (CT) scan, an ultrasound scan, and/or a magnetic resonance imaging (MRI) examination. For the current study most were referred for a CT scan (*n* = 10) or a general radiographic examination (*n =* 10).

The findings of the study are presented according to the two main approaches we followed for data analysis and interpretation. First, we presented patient descriptions related to the six elements of their imaging journey and second, the cross-cutting theme that emerged, namely person-al vulnerability.

### Elements of a patient’s imaging journey

**Fig. 2:**
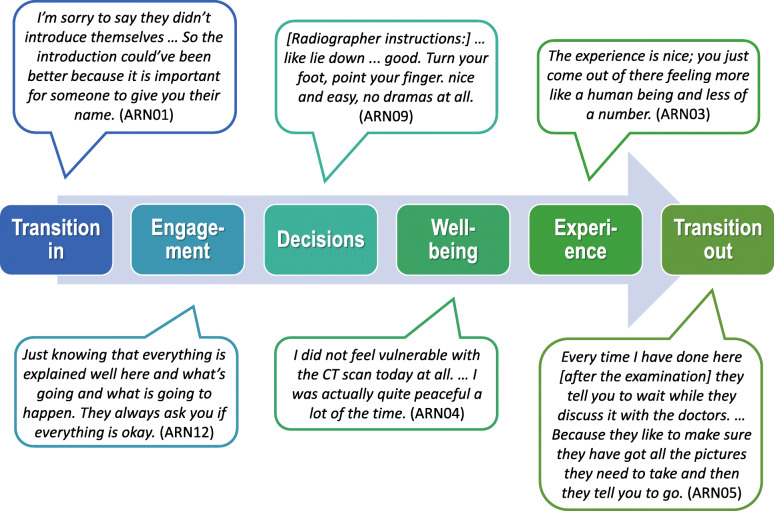
Descriptions of elements of the person-centred patient journey

Figure 2 provides a brief illustration of how a patient’s journey could unfold during an imaging investigation. Each of the six elements could be depicted as positive or negative or both.

Referral for an imaging examination was part of the initial consultation with a medical practitioner. Participants referred to this decision as an abrupt directive or a lengthier interaction: *‘They just said, “Go for an x-ray to see if there’s a fracture or not”’* (ARN14) and *‘I told him about my hands and my arms, and I also told him about my chest. So, he said, “Right, then a CAT scan and I’ll send you for a chest x-ray as well”’* (ARN17).

#### Transitioning in from the point of referral to the commencement of the examination

For some participants the transition-in phase started with an appointment – *‘When I rang and made the appointment, I was given more information’* (ARN16). The first point of contact with the imaging department was at reception, with an administrator or a radiographer. This was where a *‘personal connection’* (ARN01) could have made a big difference in putting a patient at ease.

*‘Just a case of you guys, just a smile, or a hello, or a how-are-you-this-morning. I know it’s probably part of the training, but it’s just to make people feel more comfortable and not as anxious as going through that process’* (ARN18).

Another procedure during the transition was verification of the referral and the examination(s) required. This could include adapting the schedule to accommodate discrepancies between the patient’s interpretation and the referral request.

*‘I didn’t know I actually had two things to be done but they were good enough to do the x-ray and the CT scan straight after it. … I only had an appointment for the x-ray because I can’t read the writing on the referral. So then they were like, “Oh, you’ve got to have a CT scan as well. … We’ll just pop you through.” So I only had a 10-minute wait there’* (ARN18).

Transitioning in might entail having to wait while other patients are prioritised: *‘I was treated … as an emergency to get in … but there was a lady who had a seizure, so that took priority over me. So I guess it’s kind of like hurry up and wait’* (ARN08).

One participant related that her history of a reaction to contrast injections had influenced the initial interactions.

*‘I fill in the form … [The receptionist] comes to me because she’s going to give me a glass of drink and … [the radiographer] goes, “Don’t drink it!” … I say, “No it’s okay.” … Because every time I come, that’s the only thing I can drink, that liquid [contrast]. … [The radiographer] was so worried about it. … When I came here [before], I was a little bit dizzy and same thing, they were really worried when they see me’* (ARN12).

#### Engagement – ‘Everything is explained well here’

The elements of engagement and decisions were closely intertwined throughout the examination. Engagement entailed interactions between patient and radiographer before and during the examination. The radiographer might elicit additional information on a patient’s condition or reasons for the investigation request. Part of the engagement was to explain to the patient what to expect during the examination.

*‘There was just that metallic taste but the radiographer explained it all beforehand. When it did come, I knew that was what they were talking about. But I didn’t feel any flushes or urges to pee’* (ARN02).

Patients’ sense of being heard shows that two-way engagement was taking place:

*‘The young lady and gentleman … made me feel at ease and relaxed. I think particularly when the gentleman who put the dye in listened to me when I asked something and explained everything clearly’* (ARN16).

Some participants referred to their engagement experiences during previous imaging investigations. Many compared their first experience with the current experience. One participant felt that casual communication acted as a distraction.

*‘The first ever ultrasound or x-ray I went for, I was nervous. I had no idea what was going to happen, but … now I don’t find it weird being in there with a stranger. … It’s like the talking and taking your mind off the situation and what’s happening, like, them being there and just asking about your day and what not. I find the communication part of things makes it comfortable, not the actual thing itself’* (ARN14).

Participants also used humorous interactions for coping with their own anxiety and uncertainty.

*‘The girls [have] got to be fairly professional but we had a couple of jokes and that was fine, that was normal. … A bit of a laugh or a bit of a smile, I think that helps with the treatment. … Well, it just relaxes me more than anything. I’d had an MRI four or five months ago and they’re scary things. And I remember, for that, I deliberately went out of my way to have a few jokes with the guy who was doing it because I was a bit scared with the noise and sort of things that goes along with the MRI’* (ARN11).

#### Decisions

Patient decisions related primarily to decisions by the radiographer on how to conduct a particular examination, how to position a patient or when to repeat a procedure. Patients’ cooperation with the radiographer could be seen as a form of decision to endure the discomfort that might be part of a specific investigation – *‘As long as they get the result they want, I’m happy to go along with whatever they say’* (ARN20).

*‘When you actually have it [an MRI], it’s uncomfortable. … You sort of got to stay still. That’s the worst part about it. … It’s like you’re scared to breathe because … your chest is moving. It’s just one of those things and everybody does it differently. I just tend to hold my breath and then I go red in the face. I don’t know what it is, whether it’s the nerves or whatever’* (ARN17).

#### Well-being

Well-being refers to patient safety and comfort. Patients were not always aware of the **safety** issues that radiographers had to attend to but had an understanding of the necessity for precision that accompanied some of the instructions they received.

*‘Got to make sure you listen exactly to what they’re saying because they say take a step back a bit and then you take a step back and then you might go too far. … Yeah, it’s obvious why they’re so careful positioning, putting the crosshairs on me’* (ARN20).

Participants were also appreciative of the gentle way in which they had been treated that made them feel comfortable – *‘They were good; I have been with people who are cranky, bully or pushy’* (ARN21).

‘Jenna* … was explaining everything she was doing, careful, mindful of not causing me any grief, not that she could at the moment. She was touching my very gently and I was like, that’s alright, that’s not hurting’ (ARN01). (*pseudonym)

One participant felt her well-being had been affected because of generational changes with regard to patients’ **privacy preferences**:

*‘Back in my day, you had little things like modesty sheets. … In this day and age, it is all kind of gone, even just lying in the MRI machine, you have got your knickers on and this little thin thing, just lying there and it’s quite like I do not like this people are looking up my arse … That was so much more comfortable that you are covered’* (ARN03).

#### Experience

Experience was a more encompassing element of the diagnostic imaging journey referring to how patients felt after the examination about their experience of the imaging procedure or the general treatment they had received from staff. Study participants were able to give more nuanced descriptions of **positive and negative past experiences** of actual imaging investigations, which differed according to modality and type of examination. Their first-time negative encounters made lasting impressions.

*‘When I had an MRI on my back the first time … was very confronting. … You’re right in there and it’s right down on you. And thank God, they had cold air, because I was starting to freak and thinking, “You really got to get this together. You can’t carry on like a child.” And it was not a nice experience, but I’ve gotten better, a lot better since that last one. … It’s just claustrophobic and again it’s just like that robot voice, “We’re doing this; we’re doing that.” And I was thinking, “Oh my God, it’s over my head. I don’t like this.” And you feel trapped because you’re in a cone or this cylinder … but the cold air is really good, that’s what kept my sanity the whole time. Just keep blowing that cold air and we’ll be alright’* (ARN03).

A first-time adverse reaction to a contrast medium and the way the staff members reacted also remained in the mind of one participant.

*‘Years ago I had this problem; they didn’t ask me or anything. So they give me the injection, the dye, so I went through the machine and you know how it is. And I come out and she [the radiographer] took the pillows and she just sit me straight up [too fast] and I faint. … Then she see[s] me shaking and she say[s], “Why you shaking?” And then they give me another needle supposed to be the contrast, in 10 minutes they give me 3 contrast and … I shake more … there was nothing the hospital could do because there was too much contrast. … I never come back for 5 or 6 years but I never see again the lady or the doctor that was there. … But if I see them again, I [would] run. … I just wanted to die. And plus, my husband was outside waiting for me, and nobody, nobody told him anything’* (ARN12).

Generally, participants reported positive experiences of their **current interactions** with the radiographers conducting the examinations – *‘They’re always polite’* (ARN05). Some referred to rude behaviour in past encounters at other institutions.

*‘Oh, she was just rude. There was no warmth. Like if she cracked a smile, I think it would be the end of the world. … So she’s got a real chip on her shoulder and I’m not the only one who thinks so. … The way she communicates … , there’s just nothing’* (ARN03).

Patients’ experience was also related to their confidence in technology to keep them comfortable and informed.

*‘I’ve had scans before. I don’t get anxious in that space and I know if I did, I could press a button. … I also find that it’s amazing that we can get such critical information and to see how far technology has gone. So yeah, [the scan] doesn’t bother me at all’* (ARN01).

#### Transition out

Upon completion of an examination the results were sent to the referring doctor for discussion with the patient. Sometimes a patient waited until there was confirmation about the quality of the images before leaving the radiology practice. At the end of the diagnostic imaging journey, patients were curious and sometimes even impatient to receive the results from the referring doctor.

*‘Now I want to know what they’re seeing in there. … I’ll have to wait a week to find out what’s going on, unless I ring and nag my doctor, I suppose’* (ARN06).

Another characteristic of transitioning out of the imaging encounter was that patients intended to get on with their daily lives – *‘What is the point of worrying? Does not really add anything to your life’* (ARN04) and the specialists *‘were like it’s not affecting your health in any way, so it’s not to bother doing anything about it’* (ARN02).

### Person-al vulnerability as a cross-cutting theme

Three sub-themes emerged from the analysis of data related to vulnerability as a cross-cutting theme: vulnerability of the patient as a person; vulnerability of the healthcare professional; and people come with person-al baggage.

#### Vulnerability of the patient as a person

Most participants alluded to feelings of vulnerability during the current or previous diagnostic imaging encounters. Vulnerability was linked to uncertainty regarding what the investigation would be like, but also vulnerability in terms of what the investigation would reveal about their general health. Participants used words like the following to express their vulnerability: *‘fragile’*; *‘anxious’*; *‘scared’*; *‘nervous’*; *‘panicky’*; *‘frightened’*; *‘worried’*; *‘concerned’*; *‘fear[ful]’*; *‘confronting’*. Past negative experiences and a lack of proper explanation may have aggravated feelings of being unsafe and insecure. On the other hand, familiarity with a particular procedure put the patient more at ease.

*‘It’s okay to be told, but the experience is a different thing again. But once I’d gone through it the first time, the second time wasn’t too bad because you know what to expect’* (ARN03).

Patients needed a connection with the healthcare provider to find reassurance.

*‘It makes you feel like someone is going to look after you and I think that’s really important when you’re feeling really fragile. … You just want people to be reassuring and caring, absolutely I think that is very important. … And I would imagine most people coming in for x-rays of all sorts there would be plenty of opportunity to enquire how long it’s been or how it’s happened, just so you can get that sort of connection’* (ARN01).

#### Vulnerability of healthcare professionals

Participant narratives also pointed to areas of vulnerability for healthcare professionals. For example, patients expected a certain demeanour and composure of radiographers as professionals – *‘If you’re not polite or courteous to the patients then nobody would come here’* (ARN05). And they should be able to take the proverbial punches from patients.

*‘Your main thing with the patient is your demeanour. If you can put the patient at ease, life is simple. Admittedly you do get the arseholes, but you take it on the chin with a smile’* (ARN10).

The reality is that the nature of the technological task and the fact that radiographers are accustomed to task- and product-driven actions does not leave much room for empathetic conduct.

*‘I understand you are repeating the same thing to people and it’s really hard to not sound jaded and … robotic in what you’re saying. … This is your workplace, and you need to maintain a professional, caring manner. … And majority of the time the boys and the girls have got it, but it was just that one time that woman down there was just rude. But you’ll get that in any workplace, it’s just all part of it and … how you handle it’* (ARN03).

An exceptional level of accuracy and safety is required from health professionals conducting diagnostic imaging investigations and reporting on the results. One participant narrated occasional mishaps that demonstrated the vulnerability of these professionals – *‘I also had a friend who had an ultrasound with her thyroid, and they left the word “not” out of the report and she was about to be taken to have thyroid tests’* (ARN03). Another participant mentioned that different imaging software may not be compatible: *‘There [was] something missing between the communication there. My understanding was afterwards that … [the new specialist] couldn’t access it [the images]’* (ARN08).

#### People come with person-al baggage – ‘baggage they’ve walked in the door with’

Patients’ personal lives, health concerns and other pressures helped to shape their experience of the diagnostic imaging investigation – *‘You can’t lose sight of the fact that often people [who] are here have had a bad experience … and they’re bringing their own stuff in’* (ARN03).

*‘[I want] a result. I want to know what I’m dealing with and that I’m not a bloody hypochondriac and I’m not imagining everything. I’m starting to feel like I’m a malingerer and I don’t like it. I have visions that bloody Medicare will start chasing me with a big stick because they must just see my name go through over and over again. So maybe I got red flagged, and … other than that it’s time consuming. I was just through all this when it was happening: I was in a marriage breakdown; then my husband decided he would hang himself in a motel room on my birthday. So I’m dealing with that and you’ve got all this … and the second anniversary of my son who committed suicide two years ago. So it’s not really [a] good time, and all this is adding pressure to it’* (ARN03).

Although this study focused on patient experiences, there were two participants with a medically related background who provided some perspectives on how healthcare providers’ personal life and experience could influence their behaviour towards patients.

*‘I see the change even with the young girls, their lack of understanding of people’s problems, their lack of privacy when people are at the front counter. They just kind of don’t get it, everything is a real problem. You can’t judge everyone that comes to your counter. You don’t know what baggage they’ve walked in the door with’* (ARN03).

## Discussion

The findings of this study illustrate the value of PCC while a patient transitions in, through and out of an imaging examination. This serves as a reminder of the importance of focusing on the whole person, not only biomedical markers and technical procedures [24, 25]. The findings also show that person-centred experience of diagnostic imaging is inherently integrated and interrelated and cannot be treated in isolation from the overall medical encounter and the patient’s life world.

### Elements of person-centred care

With regard to the six elements of PCC, not much has been published specifically on the kind of care that integrates the person’s life-world into a highly technical medical environment with a focus on a task that has to be performed in minimal time [26]. A golden thread of PCC in this context is communication and the application of ‘softer skills’ [27]. Satisfaction is related to patients’ reactions to explanations that make them feel at ease and safe, and their perceptions of the response to their physical and psychological needs [28, 29].

For a smooth transition into the imaging environment, the quality of the request for a radiographic examination and the completion of the request form by the referring medical practitioner are important. When scrutinising the request, the radiographer begins with engagement. This communication involves more than passing on information; it is about building rapport, explaining what will be done and addressing patient concerns and questions in a caring and compassionate way [26, 29]. Engagement leads to the mutual understanding required for the radiographer’s decisions regarding the acquisition of optimal images and for the patient’s decision to cooperate. Patients understand that the discomfort they may experience ultimately contributes to their overall well-being, which prepares them for the transition post referral to receiving the results of the investigation and information on further treatment and management.

Feelings of participation and involvement in the imaging event can alleviate patient anxiety, enhance the patient’s trust in a diagnosis that takes the whole person into account and improve the perceived quality of care [17, 30, 31]. Person-al engagement is the pivotal link of person-centred care to person-al vulnerability and person-al well-being.

### Person-al vulnerability

Participants in our study referred to their own vulnerability using a variety of terms. These feelings were triggered by their past experiences or current uncertainty regarding what to expect of the new examination or the potential diagnosis. Vulnerability can affect anyone and makes people human. It is not confined to groups like children, the elderly, people with critical mental illness or disabled groups [17, 26]. Patients referred for an imaging examination are already in a vulnerable state as a result of illness, injury, pain, fear or anxiety [26].

In our findings, patient vulnerability was seen to emanate partly from own fears and anxieties and to be related to dignity and privacy and radiographers’ professional behaviour [26, 32]. Cooperation was linked to a positive engagement with the radiographer. Power differences between radiographers and patients and the absence of dignity [33] were evident in some of the negative past experiences cited by participants. Sellman refers to the fact that recipients of care cannot always protect themselves from harmful actions by care providers [34]. In our study, participants expressed a dependency on the radiographer to act in their best interests and to protect them from risk and harm. Some cited examples where it had not been possible to protect their vulnerability due to unexpected adverse events.

The person-al baggage of both staff and patients highlighted in our findings could be interpreted in terms of Carel’s [35] reference to the unique embodied vulnerability of patients, ‘more-than-ordinary vulnerability’ (p. 214) while at the same time the responsive vulnerability of staff is acknowledged. Patient expectations of radiographers expose the areas of their professional vulnerability.

### What is expected of professional radiographers?

Participants in our study had particular expectations of the attributes and behaviour of health professionals, including expectations of scientific knowledge and technical skills along with effective patient interaction and the ability to provide quality patient care and useful diagnostic information. These expectations are also reflected in the Australian Society of Medical Imaging and Radiation Therapy’s Guidelines for Professional Conduct for Medical Imaging Practitioners, which provide standards for the following domains: professional and ethical practice; communication, teamwork and autonomy; knowledge and understanding of medical imaging; critical thinking and evaluation; service delivery and clinical management; and lifelong learning [36].

Delivering whole-person care within an imaging encounter is a complex communication interchange between patient and practitioner and includes elements of respect and compassion [26], sensitivity to cultural and micro-cultural patient preferences [37], and the planning of care based on individual needs [17]. This requires the radiographer to develop advanced skills in the areas of perception, attention and memory as well as the emotional components of caring [26]. In our study negative patient experiences were related to feelings of helplessness in the presence of technological dominance and task-oriented actions by the radiographer without sufficient application of relational skills [37]. Ultimately, the patient’s evaluation of an imaging encounter constitutes the perceived quality of service.

The challenge for radiographers and other health professionals is to go the proverbial ‘extra mile’ for the patient (e.g. with appointment bookings) but also to be mindful of the patient’s condition and experiences beyond their control (e.g. adverse reactions to a contrast medium). Radiographers are also legally accountable for their actions and should ensure safety and protection for staff, patients, and visitors [38]. Radiographers are faced with the complexity of seamlessly placing the patient at the centre of the procedure while applying their skills in performing their technical task without compromising the optimal handling of complex equipment.

Quality improvement is currently highly prioritised by most health services and various reports refer to different approaches that could be followed in diagnostic medical imaging [39, 40]. Table 1 proposes a tool that imaging teams could use to reflect on PCC in their practice and on ways to improve it. This tool, based on the six elements of PCC, could be a useful complement to other quality improvement tools and initiatives [28, 41, 43, 47, 48].

**Table 1:** Tool for use by medical imaging teams in reflecting on person-centred care [28, 41–46]

		QUESTIONS		ACTIONS		
**ELEMENT**	**ISSUE(S) FOR DISCUSSION**	**What are we doing well?**	**What could be improved?**	**HOW?** **Actions for** **improvement**	**WHO?** **Member(s) responsible for action**	**WHEN?** **Timeline & feedback**
1. Transition in	Ease of access to services: • Resources for information (e.g. website to inform patient decisions on practice or examinations; education programmes; information leaflets) • Making and getting appointments • Navigation to point of face-to-face contact (e.g. parking, signage, web information)					
	Reception: • Staff welcome and communication • Waiting room (e.g. layout, information available, provision for emergencies) • Management of waiting times (from entry to exit)					
	Documentation and verification / record keeping: • Mechanisms in place to ensure request orders are appropriately filled • Access to old imaging examinations and reports • Pregnancy tests					
2. Engagement	• Care communication • Power relations • Space / opportunity for information exchange with patient and understanding of patient needs • Staff demeanour					
3. Decisions	• Participatory decision making • Communication about procedural decisions for getting quality images					
4. Well-being	• Ethical considerations (autonomy, dignity, respect) • Safety and protection • Patient preferences and needs • Physical environment • Psychosocial aspects (e.g. emotional well-being and support) • Socio-cultural aspects (e.g. community endeavours like educating the public) • Culturally safe environment					
5. Experience	• Professionalism • Pacing of the actual examination • Efficiency of task performance • Equitable treatment					
6. Transition out	• Ease in exiting • Ensuring safety • Reporting channels and communication of outcomes					
7. Staff-related elements for providing quality care	• Equipment and accessories (e.g. equipment function status) • Medical imaging protocols • Incident writing • Utilisation of patient data • Evaluation of patient satisfaction • Education programmes for patients and staff • Peer debriefing • Multidisciplinary team engagement (e.g. including • occupational health and safety)					
8. Organisational / Institutional component	• Institutional leadership and support • Monitoring of the quality improvement program (e.g. quality assurance, quality control of equipment and workplace environment) • Standards and compliance with authorising institutional and national regulatory authorities • Internal and external stakeholder participation • Institutional culture on safety management (e.g. error disclosure and risk management)					

### Study limitations

This study had several limitations. It was conducted in the diagnostic imaging section of a single private facility. Convenience sampling was used because there was no guidance available to enable purposive sampling. The sample did, however, include male and female participants of different ethnic origins, representing a broad age range and experience of a variety of imaging examinations. Although the findings may be transferable to similar settings, they are not generalizable. An in-depth exploration of socio-cultural aspects influencing patients’ beliefs and behaviour could have provided a richer integrated insight into how societal norms and values have an impact on an individual during an imaging examination. In this regard it is important that patients and community members are encouraged to participate in deliberations on the workability of the proposed tool (Table 1). Longitudinal studies in which patients are interviewed and professional behaviour observed in a variety of situational contexts would enable a better overview of trends in the practice of PCC. Larger studies in private and public organisations among multiple stakeholder groups could provide in-depth insights into perceptions and expectations of quality whole-person care and service delivery. Although complex and challenging, the development and implementation of recording systems that include patient-reported outcomes and perspectives would result in another type of study that could promote inclusion of PCC as part of real-world evidence [49].

## Conclusion

Including the patient’s voice can meaningfully inform the real-life quality of service delivery and best-practice care principles during the medical imaging encounter. Although imaging practitioners endeavour to create a person-al space for clients to add value to the quality of their service, the creation of this space is complex. Patients are not always in a position to judge the quality management required for the optimal functionality of imaging equipment and the existing technical protocols for guaranteeing effectiveness. The proposed reflective tool is meant to be used by the entire team and leadership involved in a radiography practice in their endeavour to integrate PCC into their service quality.

## Data Availability

The datasets generated and/or analysed during the current study are not publicly available because it was a qualitative study but are available from the corresponding author on reasonable request.
