# Correction to: Creating person-al space for unspoken voices during diagnostic medical imaging examinations: a qualitative study

**DOI:** 10.1186/s12913-021-07032-9

**Published:** 2021-09-27

**Authors:** Chandra Rekha Makanjee, Anne-Marie Bergh, Deon Xu, Drishti Sarswat

**Affiliations:** 1 Department of Medical Radiation Science, University of Canberra, University Drive, Bruce, ACT 2617 Australia; 2 Research Centre for Maternal, Fetal, Newborn and Child Health Care Strategies, University of Pretoria, Private Bag X323, Gezina, 0083 South Africa

**Correction to: BMC Health Serv Res 21, 954 (2021)**

**https://doi.org/10.1186/s12913-021-06958-4**

Following publication of the original article [1], the authors identified an error in the References section and two errors in the citations due to a typesetting error.

The reference 24 and 25 should be exchanged.

The correct reference 24 and 25 should be:

24. Entwistle VA, Watt IS. Treating patients as persons: a capabilities approach to support delivery of person-centered care. Am J Bioeth. 2013;13(8):29–39. 10.1080/15265161.2013.802060.

25. Gilmore KJ, Pennucci F, De Rosis S, Passino C. Value in healthcare and the role of the patient voice. Healthc Pap. 2019;18(4):28–35. 10.12927/hcpap.2019.26031.

In the main text of the article, two citations should be corrected:

1. The citation at the end of the following sentence should be changed from [25] to **[24]**.

The unconventional hyphenation of ‘person-al’ is an attempt to keep the focus on the person, based on their ‘best interests’ and ‘quality of life’ (p. 56) [23] and to emphasise the human capabilities of patients as persons, which, according to Entwistle and Watt, include ‘personal capabilities’ (p. 34) [24].

2. The citation at the end of the following sentence should be changed from [24, 25] to **[25]**.

This serves as a reminder of the importance of focusing on the whole person, not only biomedical markers and technical procedures [25].

The original article [1] has been corrected.
